# Novel Pharmacological Approaches for Multidrug‐Resistant Tuberculosis: Review

**DOI:** 10.1155/adpp/8849786

**Published:** 2025-11-29

**Authors:** Kassahun Dires Ayenew, Habtemariam Alekaw Habteweld, Abate Wondesen Tsige, Yihenew Sewale Bizu

**Affiliations:** ^1^ Department of Pharmacy, Asrat Woldeyes Health Science Campus, Debre Berhan University, Debre Berhan, Ethiopia, dbu.edu.et; ^2^ Department of Adult Health Nursing, Medicine and Health Science Campus, Debre Markos University, Debre Markos, Ethiopia, dmu.edu.et

**Keywords:** combination therapies, MDR-TB, novel approaches

## Abstract

**Background:**

Due to its resistance to common anti‐TB drugs, multidrug‐resistant tuberculosis (MDR‐TB) presents substantial treatment problems. Optimizing therapeutic outcomes requires individualized treatment plans that take into account patient comorbidities, medication susceptibility profiles, and past treatment history. The significance of individualized medication in the treatment of MDR‐TB is emphasized in this study.

**Methods of Review:**

Current research on tailored treatment plans for MDR‐TB is summarized in this review. It highlights how pharmacogenomics, medication sensitivity testing, and patient‐centered care can be used to customize treatment plans. The utilization of combination therapies, monitoring and adaptation techniques, and novel treatment options—such as adjuvant therapy and newer agents—are also covered in the review.

**Findings:**

Important findings show that thorough medication susceptibility testing is essential for directing wise treatment decisions. Dosage modifications based on individual metabolic responses can be informed by pharmacogenomic data. Treatment regimen adherence is improved when patients participate in decision‐making. Combination therapy involving new drugs has demonstrated potential for increasing therapeutic effectiveness while reducing the emergence of resistance. Frequent monitoring makes it possible to promptly modify therapy in response to the patient response.

**Conclusions:**

Treatment for MDR‐TB must be individualized and comprehensive due to its complexity. For individuals with MDR‐TB, therapy outcomes can be greatly enhanced while lowering the risk of further resistance by combining host‐directed therapies, pharmacological breakthroughs, and continuous patient monitoring. Enhancing customized care solutions in this difficult field of infectious illness management requires ongoing research and innovation.

## 1. Introduction

One of the biggest dangers to global development and public health is antimicrobial resistance (AMR). Bacterial AMR is thought to have contributed to 4.95 million fatalities worldwide in 2019 and been directly responsible for 1.27 million deaths. Drug‐resistant infections are mostly caused by the overuse and abuse of antibiotics in humans, animals, and plants. Countries of all income levels and geographical locations are impacted by AMR. Poverty and inequality worsen their causes and effects, with low‐ and middle‐income nations being most impacted [1, 2].

Globally, multidrug‐resistant tuberculosis (MDR‐TB) is responsible for about 3.3% of newly diagnosed cases and 18% of those that have already received treatment. To effectively tackle resistant strains, it is imperative that novel therapeutic approaches be investigated immediately in light of this concerning trend [1].

MDR‐TB necessitates creative approaches to address its rising incidence and the shortcomings of existing therapies [1, 3]. The creation of novel molecular entities and the repurposing of already‐approved medications are required due to the advent of strains resistant to the two most effective first‐line anti‐TB medications, isoniazid and rifampicin [4].

### 1.1. Understanding MDR‐TB and the Need for Novel Approaches

One of the most common infectious bacterial causes of death is still tuberculosis (TB), which is brought on by *Mycobacterium tuberculosis* (Mtb) [5, 6]. Drug‐resistant TB is a result of both patient noncompliance and the improper use of antibiotics. Because MDR‐TB strains are less susceptible to traditional therapies, they pose serious problems. The problem is made worse by the second‐line medications’ poor efficacy and related toxicity. To find new anti‐TB medications and enhance treatment results, creative methods are therefore essential [5].

Rapidly eliminating actively growing bacilli, avoiding developed drug resistance, and sterilizing host tissues to avoid relapse are the objectives of antituberculosis treatment. According to current recommendations, combination therapy should be used for at least 6 months to accomplish these objectives [6]. To direct treatment, clinical isolates from regions with a high prevalence of resistance should be regularly evaluated for first‐line drug susceptibility [6].

### 1.2. Limitations of Current Treatment Regimens

The long duration of current first‐line and second‐line TB treatment regimens, which frequently call for 6–24 months of medication, can result in treatment failure and poor patient adherence [7]. To provide shorter, more efficient treatment choices, it is essential to review alternative strategies such as drug development, repurposing, host‐directed therapies (HDTs), and creative delivery methods.

### 1.3. Drug Discovery and Development

The conventional method of finding new drugs is costly and time‐consuming. To overcome resistance, new drugs that target distinct modes of action against *Mycobacterium* TB are crucial [8]. By examining current developments in drug discovery, scientists can find intriguing ideas that may result in more potent treatments.

### 1.4. Repurposing Existing Drugs

As current medications have previously undergone thorough safety evaluations, repurposing them can greatly speed up the availability of novel treatments. Research has demonstrated that medications such as linezolid and clofazimine, which were initially created for other purposes, are effective against MDR‐TB [9, 10]. New therapeutic routes with possibly cheaper research costs and shorter durations can be found by analyzing medication repurposing tactics.

### 1.5. HDTs

To combat TB more successfully, HDTs seek to strengthen the host’s immunological response. In addition to traditional antibiotics, this method provides a supplementary approach that may result in better results and shorter treatment times [11]. To learn how to use the immune system to combat MDR‐TB, it is essential to look into HDTs.

### 1.6. Innovative Delivery Systems

The bioavailability and targeted delivery of antitubercular drugs can be enhanced by developments in drug delivery technology, such as inhalable formulations and nanoparticles [12]. By improving drug absorption and minimizing side effects, investigating these systems can help create more successful treatment plans.

### 1.7. Pharmacogenomics

To tailor TB treatment based on genetic differences that impact medication metabolism and response, pharmacogenomics is essential. Comprehending these variances can aid in customizing treatments for each patient, perhaps increasing effectiveness and reducing side effects [13]. Strategies for improving MDR‐TB management can be informed by a review of pharmacogenomic techniques.

To improve patient outcomes, advance treatment options, and address a critical public health issue, it is imperative to review certain approaches in innovative therapy for MDR‐TB. Therefore, the main aim of this review is to highlight new pharmacological strategies for MDR‐TB, including developments in HDT, drug delivery technologies, alternate treatment plans, and drug discovery.

## 2. Methods

The PubMed, Embase, Scopus, and Web of Science databases were used to conduct a thorough literature review. The search concentrated on a number of important areas: new drugs such as bedaquiline, delamanid, and pretomanid; novel treatment regimens such as bedaquiline, pretomanid, and linezolid (BPaL); repurposed compounds such as clofazimine, linezolid, and thioridazine; and HDTs such as heme oxygenase‐1 (HO‐1) inhibitors, silymarin, and immunomodulation. The review also took into account cutting‐edge delivery methods such as inhalable formulations and nanoparticles. Except for foundational research that offered crucial background knowledge, the study only considered papers within the last 13 years (2012–2025). To guarantee a thorough and pertinent analysis, primary research publications were prioritized.

### 2.1. Eligibility/Inclusion Criteria

1. Complete Reports: Research involving either human participants or verified animal models which provided detailed information on innovative pharmacological strategies.
2. Developments in Drug Discovery: Articles that describe noteworthy developments in the identification and creation of drugs that combat MDR‐TB.
3. Innovative Therapeutic Strategies: Publications that address repurposed drugs, novel drug delivery methods, HDTs, and novel treatment plans.

### 2.2. Exclusion Criteria

✓ Language Restrictions: Articles published in languages other than English were not reviewed.
✓ Access Restrictions: Primary research articles were not included if they do not offer free full‐text access.
✓ Inadequate Data: Studies that do not provide clear or adequate information were also disqualified, especially if they do not publish results.

### 2.3. Data Extraction and Synthesis Methods

The purpose of this review is to summarize the state of MDR‐TB individualized treatment approaches. It emphasizes how crucial pharmacogenomics, medication sensitivity testing, and patient‐centered care are to creating successful treatment plans. The integration of combination therapies, continuous monitoring and adaptation techniques, and new treatment options such as adjuvant therapies and innovative pharmaceutical agents are also included in the review. A narrative technique was used to synthesize the data, which made it possible to identify and highlight important themes in the literature.

### 2.4. Selection Results

✓ Number of references reviewed: 65.
✓ Number of excluded references: 20.

## 3. Advances in Drug Discovery for MDR‐TB

### 3.1. New Chemical Entities

One crucial area of research is the identification of novel chemical entities (NCEs) that target vital mycobacterial functions. As indicated in Table 1, a number of new medications that show promise for treating MDR‐TB and reducing treatment duration are undergoing clinical trials. These medications target a number of processes, including protein synthesis, energy metabolism, and cell wall formation [14].

**Table 1:** Summary of mechanisms, evidence levels, trial phases, and key limitations of new chemical entities.

S. no.	Drug	Mechanism	Evidence level	Trial phase	Key limitations
1.	Bedaquiline	Prevents *Mycobacterium tuberculosis* from producing energy by inhibiting its ATP synthase, which causes bacterial cell death (Gler et al., 2012)	High; backed by phase III studies showing better treatment results than those of conventional regimens (C209 trial)	In DR‐TB, pivotal trials have demonstrated notable efficacy, and it is approved for use (Gler et al., 2012)	Hepatotoxicity and QT prolongation risks; high expense may prevent availability in low‐resource environments (Dorman et al., 2016)
2.	Delamanid	Inhibits the production of mycolic acid, which is necessary for the bacterial cell wall, and prevents mycobacterial development (Sullivan et al., 2014)	Moderate; phase III trial data demonstrate its effectiveness when used in combination therapy (Study 204)	Approved; ongoing studies are assessing long‐term safety and efficacy	Limited efficacy and the possibility of QT prolongation when administered alone (Gonzalo et al., 2019)
3.	Pretomanid	Contributes to bacterial mortality by interfering with the formation of cell walls and producing reactive nitrogen species (Gonzalez et al., 2020)	High; shown effectiveness in the bedaquiline, pretomanid, and linezolid (BPaL) regimen; clinical trials showed high success rates	Accepted; DR‐TB patients have demonstrated notable success rates in pivotal studies (Nix‐TB study)	Limited information on long‐term safety and the possibility of drug–drug interactions (Sullivan et al., 2020)
4.	Clofazimine	Interferes with lipid production in the mycobacterial membrane to prevent development (Stark et al., 2018)	Moderate; lacks comprehensive standalone efficacy data but is utilized in combo regimens for DR‐TB	It is approved and mostly used in combination with other medications	Long half‐life that causes gastrointestinal problems, skin discoloration, and persistent negative effects (Matsumoto et al., 2018)
5.	Thioridazine	Could interfere with bacterial metabolism and activity (Singh et al., 2017)	There are not much clinical data to support its usage specifically for tuberculosis (Kwon et al., 2015)	Only in the early stages of research; not commonly used to treat TB	Limited ability to combat tuberculosis and the possibility of serious adverse effects (Matsumoto et al., 2021)

Bedaquiline: A diarylquinoline called bedaquiline prevents *M. tuberculosis* from producing energy by blocking ATP synthase [15]. In the treatment of MDR‐TB, it has demonstrated excellent effectiveness and tolerability. However, issues including QT prolongation and other cardiac events must be addressed [16]. In combination regimens, bedaquiline has demonstrated notable effectiveness in treating MDR‐TB. Increased treatment success rates have been documented in clinical trials [17]. It requires monitoring during treatment due to the hazards of hepatotoxicity and QT prolongation [18]. Although it has been included in several public health initiatives, its comparatively high cost presents challenges in environments with limited resources [19]. Administering it is usually possible, but careful side effect monitoring is necessary.

Delamanid: A nitroimidazole derivative called delamanid prevents the production of mycolic acid, which is an essential part of the cell wall of mycobacteria [9]. Its effectiveness in treating MDR‐TB in conjunction with other anti‐TB medications has been shown in clinical trials [9]. In both in vitro and in vivo trials, delamanid has demonstrated strong action against *Mycobacterium* TB that is both drug‐susceptible and drug‐resistant [9]. Clinical data support the use of delamanid as part of combination therapy for MDR‐TB [20]. It has a comparable safety profile to bedaquiline, except there is a chance of hepatotoxicity and QT prolongation. It is costly, which restricts access in nations with lower incomes [19]. Usability is possible, but safety considerations necessitate supervision.

Pretomanid: Another nitroimidazole has the potential to shorten TB treatment regimens. It is often used in combination with bedaquiline and linezolid in the BPaL regimen for treating highly drug‐resistant TB [8]. It proved to be highly effective in treating MDR‐TB when used in conjunction with the BPaL regimen [21]. Peripheral neuropathy and myelosuppression are risks, particularly when taken with additional medications such as linezolid. It has high cost, comparable to other new agents, and access issues in environments with limited resources. Within organized treatment programs, it is feasible, but it needs close supervision [22].

### 3.2. Repurposing Existing Drugs

Finding novel treatments for MDR‐TB can be accelerated and made more affordable by repurposing existing medications [23, 24]. Numerous medications that were first created to treat different ailments have demonstrated possible anti‐TB properties [23].

Clofazimine: The promise of clofazimine, a Rimini dye used to treat leprosy, in the treatment of MDR/XDR‐TB is being reexamined. It is an intriguing contender because of new discoveries about its antibacterial and anti‐inflammatory properties, as well as cutting‐edge drug delivery methods [23]. However, its side effects, which include neurological, gastrointestinal, dermatological, and hematologic problems, restrict its use. There is minimal information on its effectiveness when used alone; however, it works well in combination regimens for MDR‐TB. Although the medication is usually well‐taken, it may result in gastrointestinal problems and skin discoloration. It is more accessible because it is less costly than more recent agents. It is extensively utilized in many TB treatment programs, although its lengthy half‐life may cause long‐lasting adverse effects [3, 25, 26].

Thioridazine: Thioridazine is an antipsychotic drug that has shown promise against MDR/XDR‐TB in vitro and ex vivo, as well as in mice infected with MDR‐TB and humans with XDR‐TB [23]. More study is necessary as there are insufficient data to support its use specifically for TB; its primary usage as an antipsychotic carries a risk of serious adverse effects [27].

## 4. Innovative Drug Delivery Systems

### 4.1. Nanoparticle‐Based Drug Delivery

When it comes to treating MDR‐TB, nanotechnology presents interesting ways to get beyond the drawbacks of conventional drug delivery methods [12]. Drug delivery techniques based on nanoparticles can increase bioavailability and allow for regulated and targeted drug release, especially for intracellular delivery to macrophages and pulmonary targeting [12].

Improved Bioavailability: As described in Table 2, nanoparticles can enhance the solubility and absorption of anti‐TB drugs, leading to higher drug concentrations at the site of infection [3]. Targeted Delivery: Nanoparticles can be designed to specifically target infected macrophages in the lungs, maximizing drug efficacy and reducing systemic toxicity [12].

**Table 2:** Summary of mechanisms, evidence levels, trial phases, and key limitations of nanoparticles and inhalable formulations.

S. no.	Category	Mechanism	Evidence level	Trial phase	Key limitations
1.	Nanoparticle	Distribution methods allow for targeted distribution to infected regions while improving drug solubility and bioavailability (Zhang et al., 2020; Omoteso et al., 2025)	Moderate; preclinical research is encouraging, but there has not been much clinical use of it yet (Zhang et al., 2020; Omoteso et al., 2025)	Preclinical mostly; continuous investigation into human‐useful formulations (Omoteso et al., 2025; Zhang et al., 2020)	Regulatory obstacles, stability problems, and intricate production procedures (Omoteso et al., 2025; Zhang et al., 2020)
2.	Inhalable formulations	To increase local concentrations and decrease systemic exposure, permit direct drug delivery to the lungs (Mason et al., 2019)	Moderate; more validation is necessary despite several early‐phase trials demonstrating efficacy (Mason et al., 2019)	There are ongoing phase I/II trials for several formulations (Mason et al., 2019; Omoteso et al., 2025)	Issues with formulation stability, possible respiratory tract irritation, and patient compliance. (Mason et al., 2019; Omoteso et al., 2025)

Controlled Release: By extending the duration of action and lowering the frequency of dosage, nanoparticles can offer sustained medication release [12].

### 4.2. Inhalable Formulations

A new strategy for improving medication efficacy by avoiding drug resistance mechanisms is the creation of inhalable formulations [12]. As presented in Table 2, drugs can be delivered directly to the lungs via inhaled formulations, avoiding systemic exposure and producing high local concentrations [12, 13].

Dry Powder Inhalers (DPIs): Because DPIs are more stable, they are increasingly being used to deliver biologics to the lungs [13]. Aerosol performance is improved by particle engineering techniques such as spray freeze drying (SFD) [13].

Nebulizers: Rapid and effective drug delivery is possible with nebulizers, which can administer liquid anti‐TB medication formulations straight to the lungs [13].

### 4.3. HDTs

By improving immune‐mediated Mtb clearance, HDTs offer a novel strategy for treating tuberculosis [14, 28] [29]. HDTs may stop the emergence of MDR‐TB and XDR‐TB by focusing on the host defense mechanisms rather than the microorganism [29].

### 4.4. Immunomodulation

In the fight against MDR‐TB, immunotherapies such as cytokine modulation and innovative TB vaccines provide supplementary tactics to the use of antibiotics [12].

Cytokine Modulation: It is possible to improve *M. tuberculosis* killing and decrease inflammation by modifying the host’s immune response with cytokines [12]. Preliminary studies show that aerosolized and intramuscular IFN‐γ can improve sputum conversion rates and chest radiograph outcomes in MDR‐TB patients. The effects of IL‐12 on MDR‐TB patients were examined in a clinical experiment. Bacterial clearance improved by 60% in patients receiving IL‐12 [30]. The impact of recombinant IFN‐γ on individuals with latent TB who are at high risk of acquiring MDR‐TB was evaluated in a randomized controlled experiment. When compared to the placebo group, the therapy group experienced a 50% decrease in the development of active TB [31]. The effects of TNF‐α inhibitors in patients with MDR‐TB were examined in a cohort study. Patients treated with TNF‐α inhibitors had a statistically significantly higher treatment success rate (70%) than the control group (40%) in a cohort study [32].

TB Vaccines: To strengthen the host’s immune system and offer sustained defense against TB infection, new TB vaccines are being created [12]. According to a meta‐analysis, BCG vaccination has a statistically significant preventive effect by lowering the chance of acquiring MDR‐TB by 30% (95% CI: 15%–45%), whereas there is significant variation depending on demographic and regional factors. New TB vaccines that may offer greater defense against drug‐sensitive and drug‐resistant strains of Mtb are being researched [14].

### 4.5. HO‐1 Inhibition

When used in conjunction with anti‐TB treatments, inhibition of HO‐1, an enzyme involved in heme metabolism, has demonstrated promise in lowering lung bacillary burden. Although encouraging, HO‐1’s dual function in tissue injury and protective immunity makes treatment approaches more difficult [14].

Stannsoporfin (SnMP): In mouse models, the new HO‐1 inhibitor SnMP has shown adjunctive HDT efficacy when used in conjunction with MDR‐TB treatments [14].

### 4.6. Silymarin

Milk thistle contains a flavonoid compound called silymarin, which has garnered attention because of its hepatoprotective, anti‐inflammatory, antioxidant, and anticancer effects [33]. According to recent research, silymarin may improve treatment when used with isoniazid and other first‐line anti‐TB medications [33]. To determine its involvement, more research is necessary due to the paucity of clinical data regarding its effectiveness in treating tuberculosis.

## 5. Alternative Treatment Regimens

### 5.1. Shortened Treatment Regimens

One of the main objectives of MDR‐TB treatment is to shorten its length in order to increase patient compliance and lower the possibility of side effects [34, 35]. The effectiveness and safety of shorter MDR‐TB treatment regimens are being assessed in a number of clinical trials [34].

BPaL Regimen: The BPaL regimen has shown promising results in treating highly drug‐resistant TB [34].

Fluoroquinolone‐Containing Regimens: Fluoroquinolones, such as moxifloxacin and levofloxacin, have bactericidal activity against *M. tuberculosis* and are being tested in regimens to shorten TB treatment [36].

### 5.2. Individualized Treatment Approaches

Approaches to personalized medicine that make use of host and pathogen genomic profiling show promise in reducing drug resistance and improving treatment plans [12].

Genotypic and Phenotypic Drug Susceptibility Testing: To create individualized regimens and enhance results, comprehensive genotypic and phenotypic medication susceptibility testing is required [36].

Pharmacogenomics: Optimizing drug dosage and reducing side effects can be achieved by knowing the genetic variables that affect drug metabolism and response [12]. As research in this field progresses, personalized medicine approaches could revolutionize the treatment of drug‐resistant tuberculosis by customizing treatments based on individual genetic profiles, increasing efficacy while reducing side effects, especially in patients with comorbidities or those at risk for severe side effects [12].

### 5.3. Challenges and Future Directions

Despite significant advances in pharmacological approaches for MDR‐TB, several challenges remain [31]. These include the following:

Drug Resistance Mechanisms: Developing novel medications that can overcome drug resistance requires an understanding of the molecular mechanisms causing drug resistance [12].

Limited Central Nervous System (CNS) Penetration: Many anti‐TB drugs have poor penetration into the CNS, making it difficult to treat tuberculous meningitis (TBM) [8].

### 5.4. Adverse Effects

Significant side effects are linked to a number of anti‐TB medications, which restrict their usage and affect patient compliance [10].

Lack of Biomarkers: One major obstacle to long‐term success with DR‐TB treatment is the absence of biomarkers to track response [35].

### 5.5. Future Research Directions Should Focus on

✓ Combating current and emerging drug resistance requires the development of new medications with unique mechanisms of action [6].
✓ Enhancing Medication Delivery Systems: Inhalable and nanoparticle‐based formulations can lower toxicity and increase medicinal efficacy [3].
✓ Finding and Approving Novel HDTs: HDTs have the potential to boost the host’s immune system and enhance the effectiveness of treatment [28, 29, 33].
✓ Optimizing Treatment Plans: Personalized and condensed treatment plans can decrease therapy duration and increase patient compliance [34, 35].
✓ Creation of Biomarkers to Track Response to Treatment: Biomarkers can be used to determine which patients are responding to treatment and which require alternative therapies [35].

## 6. Economic and Implementation Considerations in Low‐Resource TB‐Endemic Regions

### 6.1. Public Health Implications

Public health systems are greatly impacted by the growth of MDR‐TB, especially in low‐resource environments where TB is endemic. Increased morbidity and death from high MDR‐TB rates can put further demand on the already scarce healthcare resources. In addition to cutting‐edge treatments, effective public health initiatives such as contact tracing, early detection, and extensive treatment programs are necessary for the management of MDR‐TB.

### 6.2. Accessibility of Novel Therapies

Although new treatments such as bedaquiline appear promising, it is crucial to consider their accessibility in environments with limited resources. The availability of novel medications may be restricted by their high cost, particularly in nations with limited healthcare resources. For example, many national TB programs are still hindered by the cost of bedaquiline. Investigating methods for lowering medication costs is therefore crucial. These include promoting generics, negotiating rates with pharmaceutical corporations, and making use of international financing sources.

### 6.3. Cost‐Effectiveness Analysis

To ascertain the economic feasibility of innovative treatments in low‐resource environments, comprehensive cost‐effectiveness evaluations must be conducted in conjunction with their implementation. When looking at long‐term health outcomes, research shows that the incremental cost‐effectiveness ratio (ICER) of implementing novel treatments, such as bedaquiline, can be advantageous when compared to conventional regimens [8]. To ensure that scarce resources are distributed efficiently, policymakers should give priority to actions that offer the most value for the money.

### 6.4. Risks of Resistance Development

Concerns regarding the development of resistance are raised by the introduction of novel medications such as bedaquiline. According to studies, resistant strains can arise as a result of inappropriate use or insufficient treatment regimens [8]. Setting strict rules for the application of novel treatments and keeping a careful eye on resistance trends are essential. The risk of resistance can be reduced by putting in place stewardship initiatives that support responsible prescribing practices.

### 6.5. Community Engagement and Education

For novel TB treatments to be implemented successfully, community involvement is essential. Improving outcomes and lowering the chance of resistance development can be achieved by educating patients and communities about the significance of following treatment plans and the dangers of not finishing therapy. Locally specific public health initiatives can raise knowledge and encourage acceptance of new treatments.

## 7. Conclusion

A multifaceted strategy that takes into account accessibility, infrastructure development, community involvement, and economic considerations is needed to address the difficulties presented by MDR‐TB in low‐resource environments. Stakeholders can create holistic plans that enhance treatment results and fortify public health systems in TB‐endemic areas by tying these wider implications to reviewed data on innovative medicines.

## 8. Recommendations

### 8.1. For Clinicians

1. Adopt Guidelines Based on Evidence: Keep abreast of the most recent clinical recommendations and data pertaining to the application of innovative treatments such as bedaquiline, delamanid, and other more recent substances.
2. Stress Adherence: Create plans to improve patients’ compliance with MDR‐TB treatment plans. Directly observed therapy (DOT), patient education regarding the significance of finishing treatment, and addressing adherence hurdles are a few examples of this.
3. Keep an Eye out for Negative Effects: Keep a close eye on patients to look for any negative effects from new treatments. Treatment success rates and patient outcomes can be enhanced by early detection and control of side effects.

### 8.2. For Researchers

1. Perform Pragmatic Trials: We put your attention on carrying out practical research that assesses the safety and efficacy of innovative treatments in a range of populations, especially in low‐resource environments where MDR‐TB is most common.
2. Examine Combination Therapies: To improve efficacy and lower the risk of resistance, we examine the possible advantages of combination therapies that include new drugs in addition to currently used treatments.
3. Evaluate Long‐Term Outcomes: Research the quality of life, relapse rates, and effects on public health indicators such as transmission rates of patients treated with innovative medicines.

### 8.3. For Policymakers

1. Ensure Drug Accessibility supports laws that facilitate the availability of reasonably priced, innovative TB treatments. This might entail promoting the development of generics and haggling over prices with pharmaceutical companies.
2. Invest in Healthcare Infrastructure provides funds to fortify healthcare systems in areas where tuberculosis is endemic. This includes expanding diagnostic laboratory capabilities and strengthening healthcare worker training initiatives.
3. Encourage Public Health Campaigns provides financial support for projects that increase community knowledge of MDR‐TB and the significance of treatment adherence and involves stakeholders and local leaders to promote involvement and trust.

## Ethics Statement

The authors have nothing to report.

## Consent

The authors have nothing to report.

## Disclosure

All authors have approved the final version.

## Conflicts of Interest

The authors declare no conflicts of interest.

## Author Contributions

Kassahun Dires Ayenew and Yihenew Sewale Bizu conducted the review. Habtemariam Alekaw Habteweld and Abate Wondesen Tsige commented on the review.

## Funding

The authors received no specific funding for this work.
